# Chronic granulomatous disease, the McLeod phenotype and the contiguous gene deletion syndrome-a review

**DOI:** 10.1186/1476-7961-9-13

**Published:** 2011-11-23

**Authors:** Casey E Watkins, John Litchfield, Eunkyung Song, Gayatri B Jaishankar, Niva Misra, Nikhil Holla, Michelle Duffourc, Guha Krishnaswamy

**Affiliations:** 1 Quillen College of Medicine, East Tennessee State University, Johnson City, Tennessee, USA; 2 Department of Medicine, Quillen College of Medicine, East Tennessee State University, Johnson City, Tennessee, USA; 3 Department of Pediatrics, Quillen College of Medicine, East Tennessee State University, Johnson City, Tennessee, USA; 4 Department of Pharmacology, Quillen College of Medicine, East Tennessee State University, Johnson City, Tennessee, USA; 5 Division of Allergy, Asthma and Clinical Immunology, Department of Medicine, Quillen College of Medicine, East Tennessee State University, Johnson City, Tennessee, USA

**Keywords:** Granulomatous Disease, Chronic, gene deletion, XK Kell blood group precursor (McLeod phenotype), human, KX antigen, human, anemia, hemolytic

## Abstract

Chronic Granulomatous Disease (CGD), a disorder of the NADPH oxidase system, results in phagocyte functional defects and subsequent infections with bacterial and fungal pathogens (such as *Aspergillus *species and *Candida albicans*). Deletions and missense, frameshift, or nonsense mutations in the gp91^phox ^gene (also termed CYBB), located in the Xp21.1 region of the X chromosome, are associated with the most common form of CGD. When larger X-chromosomal deletions occur, including the XK gene deletion, a so-called "Contiguous Gene Deletion Syndrome" may result. The contiguous gene deletion syndrome is known to associate the Kell phenotype/McLeod syndrome with diseases such as X-linked chronic granulomatous disease, Duchenne muscular dystrophy, and X-linked retinitis pigmentosa. These patients are often complicated and management requires special attention to the various facets of the syndrome.

## Introduction

Primary immune deficiencies can involve defects in phagocyte function, resulting in Chronic granulomatous disease (CGD) [1,2]. CGD is characterized by repeated infections with bacterial and fungal pathogens, as well as the formation of granulomas [1-4]. In this disease, the NADPH oxidase system is dysfunctional due to specific gene mutations, culminating in an inability of the phagocyte to eliminate pathogenic organisms. Typically, this defect in phagocyte function leads to serious infections including *Staphylococcus aureus*, *Pseudomonas *species, *Nocardia *species, fungi such as *Aspergillus *species, and *Candida albicans*. Significant morbidity and mortality may result.

The genetic defect in CGD can be transmitted in either an X-linked or autosomal recessive manner [1,5-7]. Genes regulating NADPH assembly and function are most commonly affected. When X-linked disease occurs, deletion of the contiguous genes that border the gene on the X chromosome that regulates NADPH function can result in the "contiguous X-chromosome gene deletion syndrome" [8-12]. Mutations extending into these surrounding genes may result in Duchenne muscular dystrophy (DMD) or Retinitis pigmentosa [13,14]. Other conditions, such as the McLeod syndrome, may also result in such patients [15-18]. In some selected situations, mutations in other innate immune system genes such as the complement pathway genes (especially mannose binding lectin) may further complicate disease severity and management [19].

### CGD and the Contiguous Gene Deletion Syndrome

X-linked CGD is responsible for the majority (> 60%) of the CGD cases seen in the United States [2,20]. The X-linked type is caused by a mutation in the gp91^phox ^gene, which is also referred to as the cytochrome b-245 beta polypeptide (CYBB) gene. Autosomal recessive CGD is seen in the remaining 35% of cases and arises due to mutations of the other components of the NADPH oxidase system [1,2,5-7]. These mutations include p22^phox ^, p67^phox^, and p47^phox ^. Of these, the dominant mutation observed is in the p22^phox ^gene which accounts for almost 25% of the autosomal recessive cases. The associated phenotypes are also referred to as the A22/A47/A67 CGD. The phenotype of the gp91^phox ^gene mutation is referred to as X-CGD. Mutations of other nearby genes, p40^phox ^and Rac, have yet to be associated with any CGD phenotype [2,20]. Gene location and associated disease state are described in Table 1.

**Table 1:** Gene and Chromosomal localization

Condition	Gene	Chromosome	Comments	Clinical
**X-linked CGD**	CYBB	XP21	Deletion or missense, nonsense and/or frameshift mutations may occur	Opportunistic infection Autoimmunity Organ dysfunction
**McLeod syndrome**	XK	XP21	Absent Kx antigen and weak expression of Kell on RBC surface XK is linked to Kell blood group antigen Kell locus mutations can also lead to the syndrome	Acanthocytosis Elevated CPK Huntington's chorea-like disease Muscle weakness and atrophy Cardiomyopathy Psychiatric disease Cognitive impairment

**Figure 1:**
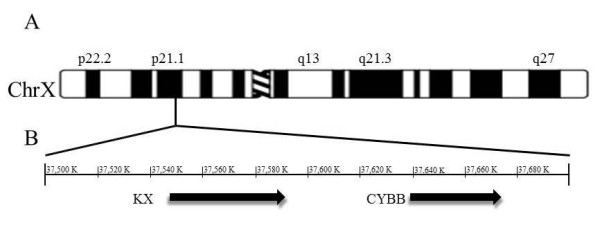
**XK and CYBB are neighboring genes**. A. Idiogram of human chromosome X at 550 banding resolution. The bar indicates the location XK and CYBB genes and is expanded below to show the genomic region. B. Genomic context of Xp21.1. The region of chromosome X from 37,500 to 37,700 K is shown. Arrows indicate the location of the XK and CYBB genes. Numbering is based upon GenBank accession number NC_000023.10.

**Figure 2:**
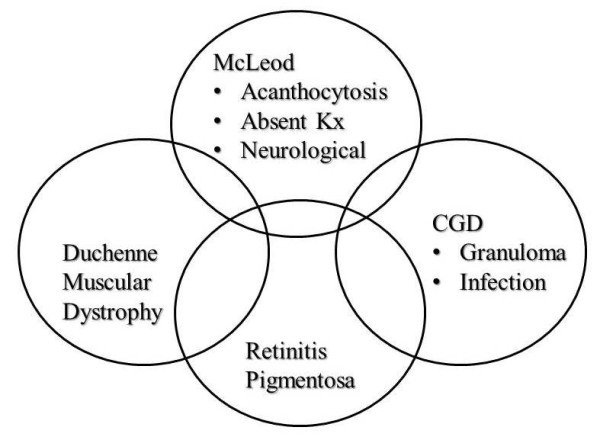
**Complicating conditions often seen in the Contiguous gene deletion syndrome associated with chronic granulomatous disease**.

The CYBB gene spans a 30 kb region in the Xp21.1 region of the X chromosome (Figure 1A). Deletions and frameshift, missense, nonsense, or splice site mutations of the CYBB gene can contribute to defects seen in CGD. On occasion, larger X-chromosomal deletions may occur. The Kell locus or XK gene, which encodes an essential glycoprotein for Kell antigen expression, may be involved in such cases. These larger deletions that extend across multiple genes will result in manifestations of the "Contiguous gene deletion syndrome" (Figure 1B)[8,9]. As stated earlier, this syndrome (Figure 2) encompasses the McLeod syndrome in association with X-linked Chronic granulomatous disease, Duchenne muscular dystrophy, and/or X-linked Retinitis pigmentosa [10,14,15].

### The Kell blood group and Kell antigens

The Kell blood group system was first identified by Coombs in 1946 when ABO and Rhesus incompatibility were excluded in a mother, Mrs. Kelleher, who had delivered a baby with hemolytic anemia. It is now considered the third most important blood group system based on immunological potency. The antigens of the Kell system are numerous and complex [17,21-25]. The K antigen may be the most significant antigen of the Kell system with regards to development of disease and transfusion-related complications.

**Figure 3:**
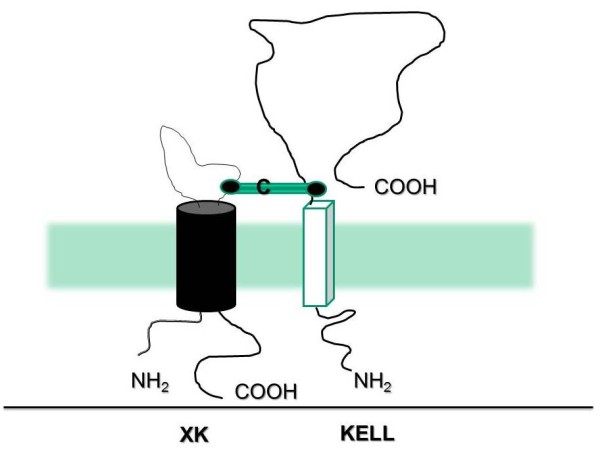
**Structure of the XK and Kell proteins**.

The Kell antigens, which are encoded for by the KEL gene, are carried by the transmembrane Kell glycoprotein (XK) (Figure 3). The KEL gene is found on chromosome 7 (7q33). It is highly polymorphic [26,27] and encodes the numerous Kell antigens. There appear to be two major co-dominant alleles referred to as k (Cellano) and K (Kell) that differ by a single amino acid change; the latter is considered the more potent immunogen.

Although there have been 25 Kell antigens identified, the "k" antigen is the most common. The phenotype K-k+ is seen in a majority of Caucasians and African Americans, while a smaller number (9%) of Caucasians may be K+k+. When the Kell antigens are absent on red blood cells, this is referred to as the rare K null phenotype (K_0_). Individuals with this phenotype produce anti-Ku antibodies that target RBCs expressing Kell antigens, but are otherwise healthy. Anti-Ku antibodies may result in the development of moderate to severe transfusion reactions following transfusion from a donor who is Kell+. For this reason, patients with the K_0 _phenotype should only be transfused with K_0 _blood products. Anti-Ku antibodies are also involved in fetomaternal immunization. Pregnant females with the K_0 _phenotype produce anti-Ku antibodies which target Kell antigens located on the surface of fetal RBCs. These antibodies are responsible for the third most common cause of hemolytic anemia in the newborn. The McLeod phenotype is seen with a severe reduction in Kell antigens; this can be associated with CGD [23-25].

### The McLeod Syndrome

The McLeod syndrome is characterized by an absence of the Kx antigen on red blood cells. This is associated with weak expression of Kell antigens. Kell antigens are covalently bound to the RBC transmembrane XK protein. When XK is absent on RBC membranes, McLeod syndrome develops. In this syndrome, the Kell antigens are weakly expressed which leads to the appearance of abnormally shaped RBCs (referred to as acanthocytes) [28]. Characteristics of the Kell and XK proteins are shown in Table 2. The McLeod syndrome manifests either as a hemolytic anemia following transfusion of Kell(+) RBC to a Kell(-) recipient or as a delayed onset (usually in the 4^th ^decade or later) of neuropsychological or cardiovascular impairment in patients with CGD.

**Table 2:** Characteristics of the Kell system

**Kell protein**	93 kDa type II membrane glycoprotein Short N-terminal intracellular segment with one cysteine residue Single transmembrane section Large extracellular domain (665 amino acids with 15 cysteine residues) One cysteine residue (Cys72) in extracellular domain binds XK protein Homology to certain zinc endoproteases Demonstrates endothelin-3 converting enzymatic activity Encoded by the KEL gene (7q33) K0 (null) RBC lack Kell antigens but have enhance XK activity KEL gene is inherited autosomally
**XK protein**	Molecular weight of 50.9 kDa 10 transmembrane segments Short N-terminal domain (intracellular) Large C-terminal domain (intracellular) A large hydrophilic loop (Figure 3) Single cysteine residue that binds covalently to Cys72 on Kell Loss of XK leads to McLeod syndrome that has X-linked inheritance

As reviewed by Jung et al., this disorder may be discovered accidentally by routine screening of apparently healthy blood donors [29]. Affected individuals may be detected by elevated creatinine kinase levels and acanthocytosis [29]. In addition to extravascular hemolytic anemia, patients manifesting the McLeod syndrome may develop multisystem disease, including splenomegaly and neurological problems late in life [30]. With time, neurological complications and manifestations of Huntington's disease-like disorder may become evident. The neurological symptoms may appear between the 2^nd ^and 6^th ^decades of life. Besides Huntington's chorea-like disease and cardiomyopathy, patients develop muscle weakness and atrophy, psychiatric disease, and cognitive impairment [16,17,29-34]. In some cases, the disorder that develops may be indistinguishable from neuroacanthocytosis with chorea, orofacial dyskinesia, dysarthria, and dementia [35]. It is therefore essential to evaluate these more complex patients for the conditions listed in Table 3 including generalized seizures and cardiomyopathy which may culminate in ventricular and atrial arrhythmia.

**Table 3:** Contiguous Gene Syndrome (including McLeod Syndrome) complicating CGD

Clinical component	Evaluation (selected)
**Myopathy**	CPK level (serum)
	EMG/NCS
**Hemolytic Anemia**	Reticulocyte cell count
	Low haptoglobin
	Acanthocytosis
**Late-onset neurological syndrome**	
Cerebral atrophy	CT/MRI
Neuropathy	EMG/NCS
Huntington's chorea-like disease	CT/MRI
Neuropsychological and cognitive impairment	Neuropsychological testing
Myopathy/DMD	EMG/NCS
	Muscle biopsy*
Seizures	EEG
**Glomerulopathy with renal failure**	Serum creatinine level
	24 hour urine analysis
	Renal imaging
	+/- kidney biopsy
**Cardiovascular disease**	
Cardiomyopathy	ECHO
Arrythmia	EPS

CPK = creatine phosphokinase; EMG/NCS = electromyography and nerve conduction study

CT = computed tomography; MRI = magnetic resonance imaging; EEG = electroencephalography

ECHO = echocardiography (transthoracic or transesophageal); EPS = electrophysiological studies

DMD = Duchenne muscular dystrophy; * = typical muscle histology of DMD and absent muscle dystrophin

### Duchenne Muscular Dystrophy

DMD is an X-linked progressive myopathy that can occasionally complicate a contiguous gene deletion syndrome involving X-linked CGD [13,34]. In the case reported by Kang et al., the DMD manifestations developed several years after the successful treatment of CGD by allogeneic stem cell transplantation [13]. This male child had a large scale deletion spanning the region between CYBB and DMD on the X chromosome. No dystrophin was expressed in the muscle, and a muscle biopsy demonstrated the typical histological changes of DMD. Dystrophin is located on the short arm of the X chromosome at the locus p21. Deletion of the dystrophin gene is commonly seen in DMD-especially when complicating CGD and the contiguous gene deletion syndrome. In other cases, point mutations and/or microdeletions may occur. Western blotting or immunocytochemistry for dystrophin protein expression can also be used diagnostically. The disease manifests as clumsy gait, development of the typical "Gower's maneuver" (the child has to place one hand on the knee in order to stand upright while rising from a seated position) and calf muscle changes (characterized by pseudo-hypertrophy). The child develops progressive muscular weakness that results in frequent falls, inability to climb and, eventually, culminates in the child becoming wheelchair bound. Cardiomyopathy and respiratory failure may result in severe morbidity and mortality. Treatment may involve physiotherapy, application of a brace, and surgery where indicated. Attempts are being made to develop pharmacological approaches (such as the use of glucocorticoids, not without adverse effects, or more muscle-specific therapies) and gene therapy to reverse the disease process.

### Retinitis Pigmentosa

This is a condition that leads to progressive visual loss due to loss of the photoreceptors. There are several reports of Retinitis pigmentosa complicating CGD and the Contiguous gene deletion syndrome [14,34,36-38]. Ophthalmic examination reveals fairly typical findings including pigment loss, waxy pallor of the optic nerve head, and attenuation of the retinal arterioles. There are several genetic variants of Retinitis pigmentosa. The X-linked type, involving mutations of the *RPGR *gene, is seen in the Contiguous gene deletion syndrome. Clinically, there is progressive loss of visual acuity and night blindness followed by a rapid decline in vision and blindness by the 4^th ^decade.

## Conclusion

CGD is a chronic disease caused by a mutation in a gene encoding essential components of NADPH oxidase function. As a result, increased susceptibility for infections occurs. The more common form of CGD involves the gene regulating NADPH function that is located on the X-chromosome. A deletion that results in X-linked CGD can also involve contiguous genes. This results in the "contiguous X-chromosome gene deletion syndrome" with manifestations of the McLeod syndrome, Duchenne muscular dystrophy and X-linked retinitis pigmentosa that further complicate disease severity and management.

## List of Abbreviations

CGD: chronic granulomatous disease; CGS: contiguous gene syndrome; RBC: red blood cell; CYBB: cytochrome b-245 beta polypeptide.

## Competing interests

The authors declare that they have no competing interests.

## Authors' contributions

CW-organized and assisted in writing manuscript; JL-assisted with manuscript review; ES-assisted in writing manuscript; GJ-assisted in writing manuscript; NM-assisted with manuscript review and corrections; NH-assisted with figure and literature review; MD-assisted with generating figure; GK-conceived of the manuscript, generated all figures and developed the format. All authors have read and approved the final manuscript.
